# Discharge Prescription of Renin-Angiotensin System Inhibitors After Hyperkalemia and Its Association With Mortality and Dialysis Outcomes

**DOI:** 10.7759/cureus.110247

**Published:** 2026-06-04

**Authors:** Yarden Weiss, Lior Cohen-Yatziv, Ariel Ben Shimol, Nimrod Adatto Levy, Ilia Beberashvili, Shai Efrati, Shani Zilberman Itskovich

**Affiliations:** 1 Department of General Practice, Gray Faculty of Medical and Health Sciences, Tel Aviv University, Tel Aviv, ISR; 2 Division of Infectious Diseases, Department of Medicine, University of Illinois, Chicago, USA; 3 Department of Internal Medicine, Shamir Medical Center, Zerifin, ISR; 4 Department of Internal Medicine, Tel Aviv Medical Center, Tel Aviv, ISR; 5 Department of Nephrology and Hypertension, Shamir Medical Center, Zerifin, ISR

**Keywords:** acei, arbs, chronic kidney disease, diabetes, heart failure

## Abstract

Background: Medication changes at hospital discharge may influence post-discharge outcomes. Renin-angiotensin system inhibitors (RASi) are frequently discontinued during hospitalization for hyperkalemia; however, guidance regarding discharge prescribing is limited. We evaluated the association between RASi prescription at discharge and post-discharge outcomes in patients hospitalized with hyperkalemia.

Methods: In this single-center retrospective cohort study at a large tertiary hospital, adults admitted between 2005 and 2020 with a potassium level > 5.5 mEq/L while receiving chronic RASi therapy were included and followed through 2022. Exposure was defined as prescription versus non-prescription of RASi at discharge. Propensity score matching was used among survivors of the first hospitalization to balance baseline characteristics. Kaplan-Meier and multivariable Cox models were used to assess all-cause mortality and chronic dialysis.

Results: Among 12,326 patients with confirmed hyperkalemia, 9,051 survived the index hospitalization; 4,388 were included after matching (2,194 per group). Prescription of RASi at discharge was associated with lower mortality (401 (18.3%) vs 642 (29.3%); hazard ratio (HR), 0.62; 95% confidence interval (CI), 0.55-0.71) but a higher risk of chronic dialysis initiation (167 (8.2%) vs. 111 (5.7%); HR, 1.57; 95% CI, 1.23-1.99).

Conclusions: In patients hospitalized with hyperkalemia, discharge prescription of RASi was associated with lower mortality but a higher incidence of chronic dialysis initiation. Dialysis initiation may reflect both progression of renal disease and longer survival among patients discharged on RASi therapy. Given the observational study design, these findings should be interpreted as associations rather than causal effects and underscore the complex risk-benefit considerations surrounding discharge medication decisions.

## Introduction

Renin-angiotensin system inhibitors (RASi) are cornerstone therapies in the management of chronic conditions such as heart failure, hypertension, and chronic kidney disease (CKD) [1-3]. These agents have been shown to reduce mortality and hospitalizations among patients with heart failure and to slow the progression to end-stage kidney disease in individuals with CKD or diabetes [1,3,4]. Furthermore, RASi are endorsed by major health organizations as a first-line treatment for hypertension, with demonstrated benefits in reducing both morbidity and mortality [2]. Despite their well-established efficacy, RASi are frequently discontinued during hospitalizations [5].

One of the most significant adverse effects of RASi is RASi-associated hyperkalemia, defined as a serum potassium level greater than 5.5 mEq/L. Patients receiving RASi are often predisposed to hyperkalemia due to underlying conditions such as CKD, congestive heart failure (CHF), or diabetes mellitus (DM) [6,7]. Hyperkalemia is commonly categorized by severity: mild (5.5-5.9 mEq/L), moderate (6.0-6.4 mEq/L), and severe (>6.5 mEq/L) [8] and may range from asymptomatic laboratory abnormalities to severe life-threatening arrhythmias [7,9].

The management of hyperkalemia during hospitalization remains a subject of ongoing debate, and a significant gap persists between clinical guidelines and real-world practice, particularly in the context of RASi-associated hyperkalemia [10]. In cases of moderate to severe hyperkalemia, up to 50% of patients receiving the maximal RASi dose experience dose reductions or complete discontinuation, while approximately one-third of those on submaximal doses have their treatment halted entirely [10]. Dose reduction or discontinuation of RASi has been consistently associated with worse clinical outcomes in patients with CHF and CKD [10-12]. The challenge of managing RASi-associated hyperkalemia has led to the emergence of new therapeutic strategies in recent years aimed at reducing discontinuation of RASi therapy, including the use of novel potassium binders such as patiromer and sodium zirconium cyclosilicate [8].

Despite evolving guideline recommendations and the availability of newer potassium-lowering therapies [13,14], real-world guidance regarding continuation versus discontinuation of RASi therapy at hospital discharge following hyperkalemia remains limited. This study aimed to investigate the association between discharge prescription versus non-prescription of RASi therapy following hospitalization with RASi-associated hyperkalemia and post-discharge survival. Secondary outcomes included chronic dialysis initiation following discharge and time to mortality or dialysis initiation. In addition, subgroup analyses were performed to evaluate whether RASi discontinuation among patients with hypertension and diabetes, in the absence of CKD or CHF, was associated with mortality.

## Materials and methods

Study design and data collection

This retrospective cohort study was conducted at the Tel Aviv Sourasky Medical Center (Ichilov Hospital), Tel Aviv, Israel, between January 2005 and December 2020, with follow-up data collected through 2022. This study was approved by the Institutional Ethics Committee of Tel Aviv Sourasky Medical Center (approval number: TLV-0356-22) before its initiation. Due to the nature of the retrospective study, informed consent was waived.

Data were extracted from the "Chameleon" electronic medical record system, which integrates comprehensive patient-level clinical data. Extracted variables included demographic characteristics, comorbidities including Charlson comorbidity scores, medication lists, laboratory results, and dialysis-related data. Post-discharge outcomes were retrieved through a national registry linked to the Israeli Ministry of the Interior and integrated within the "Chameleon" system. This nationwide registry provides routinely updated and government-monitored data regarding mortality and chronic dialysis initiation, allowing comprehensive follow-up and capture of outpatient deaths and dialysis outcomes.

Patients

The study population included adult patients (≥18 years) who were hospitalized for any cause and were found to have documented hyperkalemia, defined as a serum potassium level > 5.5 mEq/L, while receiving chronic angiotensin-converting enzyme inhibitors (ACEi) or angiotensin receptor blockers (ARBs) therapy for at least three months before hospitalization. All classes and doses of ACEi and ARBs were included. Patients with borderline hyperkalemia (5.2-5.4 mEq/L) were excluded, as these values are typically not associated with changes in chronic medication regimens. Patients with pseudohyperkalemia, i.e., patients with high hemolysis indices but normal follow-up potassium levels, were excluded. Eligibility was limited to patients with complete and available medical records and laboratory data from the electronic health record system used during the study period. Patients were included in only one cohort at their first hospitalization, if eligible. As part of the data quality assurance, a representative sample of 900 cases was cross-validated against original medical records using the "Chameleon" system.

Patients included in this study were divided into two groups: those prescribed RASi therapy at discharge and those not prescribed RASi therapy at discharge. Changes in dosing were not captured; only the prescription of RASi at discharge was recorded.

To evaluate the continuation or reinitiation of RASi following the first hospitalization, we conducted a follow-up analysis among patients who survived their first admission. Medication data were extracted for all patients who were subsequently readmitted to Tel Aviv Sourasky Medical Center during the study period and had a documented medication list available. For each patient, we identified whether RASi therapy appeared on the active medication list at the time of subsequent admission. Continuation rates were compared between patients who were prescribed RASi at discharge from the first hospitalization and those who were not.

Outcomes

Clinical outcomes, including all-cause mortality and initiation of dialysis, were compared between the two study groups. The primary outcome was all-cause mortality. Secondary outcomes included time to death, initiation of chronic dialysis, and time to chronic dialysis initiation. Time to event was defined as the number of days from hospital discharge to death or to first dialysis initiation. Acute dialysis was defined as dialysis initiated during hospitalization in patients without end-stage kidney disease that did not continue after discharge. Chronic dialysis initiation was defined as dialysis that persisted following hospitalization without discontinuation.

Laboratory measurements

Serum potassium, creatinine, and other biochemistry results were obtained from the hospital’s central laboratory database. All tests were performed on serum samples using automated analyzers (Roche Cobas platforms) with routine internal quality control. Hyperkalemia classification was based on the highest potassium value measured at admission or within the first 24 hours. Cases of pseudohyperkalemia were excluded when a high hemolysis index was documented and follow-up potassium levels were normal.

Statistical analysis

Statistical analyses were conducted using the R statistical software (2025), ​​​​​​version 4.5.1 (R Foundation for Statistical Computing, Vienna, Austria). Propensity score matching (PSM) was performed at the outset to minimize baseline confounding between patients discharged with and without RASi prescription. Patients who died during their first hospitalization were excluded before performing PSM and subgroup analysis. Thus, the PSM procedure was applied only to patients who survived to discharge to ensure that matching reflected the baseline characteristics of the population eligible for outpatient RASi prescription or discontinuation.

Matching was performed using multivariable logistic regression; patients were matched 1:1 using the nearest-neighbor algorithm without replacement based on clinically relevant covariates such as age, gender, potassium levels, creatinine, CKD, CHF, DM, history of myocardial infarction (MI), and Charlson comorbidity index. CKD was defined as patients with a diagnosis of CKD before first hospitalization according to ICD-9 codes. Post-matching balance was assessed using standardized mean differences (SMDs), with values < 0.1 considered indicative of adequate balance, in accordance with accepted methodological standards. No predefined caliper was applied.

Continuous variables were reported as means ± standard deviation (SD) or medians with interquartile ranges (IQRs), depending on data distribution. Comparisons of continuous variables were performed using paired t-tests or Wilcoxon signed-rank tests, as appropriate. Categorical variables were compared using Chi-square tests or Fisher’s exact test. Odds ratios (ORs) were estimated using multivariable logistic regression models, whereas hazard ratios (HRs) were derived from Cox proportional hazards regression models for time-to-event outcomes, for all models. Interaction terms were included in regression models to evaluate effect modification in predefined subgroup analyses.

Time-to-event outcomes, including (i) time to death and (ii) time to dialysis initiation, were evaluated using Kaplan-Meier survival curves and a log-rank test. Cox proportional hazards models were used to estimate adjusted HRs for each outcome, including covariates such as treatment group (RASi vs. no RASi), with additional adjustment for age and gender to account for potential residual imbalance after PSM. Proportional hazards assumptions were evaluated using Schoenfeld residual testing and graphical inspection of Schoenfeld residual plots. Time-to-event analyses were anchored at discharge from the index hospitalization for hyperkalemia. To account for the competing risk of death before dialysis initiation, a Fine-Gray competing-risk regression analysis was performed in the propensity score-matched cohort, with dialysis initiation defined as the event of interest and death treated as a competing event. Subdistribution hazard ratios (sHRs) with 95% confidence intervals (CIs) were calculated.

Missing laboratory data (e.g., creatinine) accounted for less than 3% of observations and were handled using median imputation to preserve sample size. All statistical tests were two-sided, and p-values < 0.05 were considered statistically significant.

Subgroup analyses were conducted according to age, sex, CKD, DM, CHF, Charlson comorbidity score, and potassium level and were assessed following PSM. The results are presented as HRs with 95% CIs in a forest plot. Given the exploratory nature of the subgroup analyses and the observational design of this study, formal corrections for multiple comparisons were not applied.

## Results

**Figure 1:**
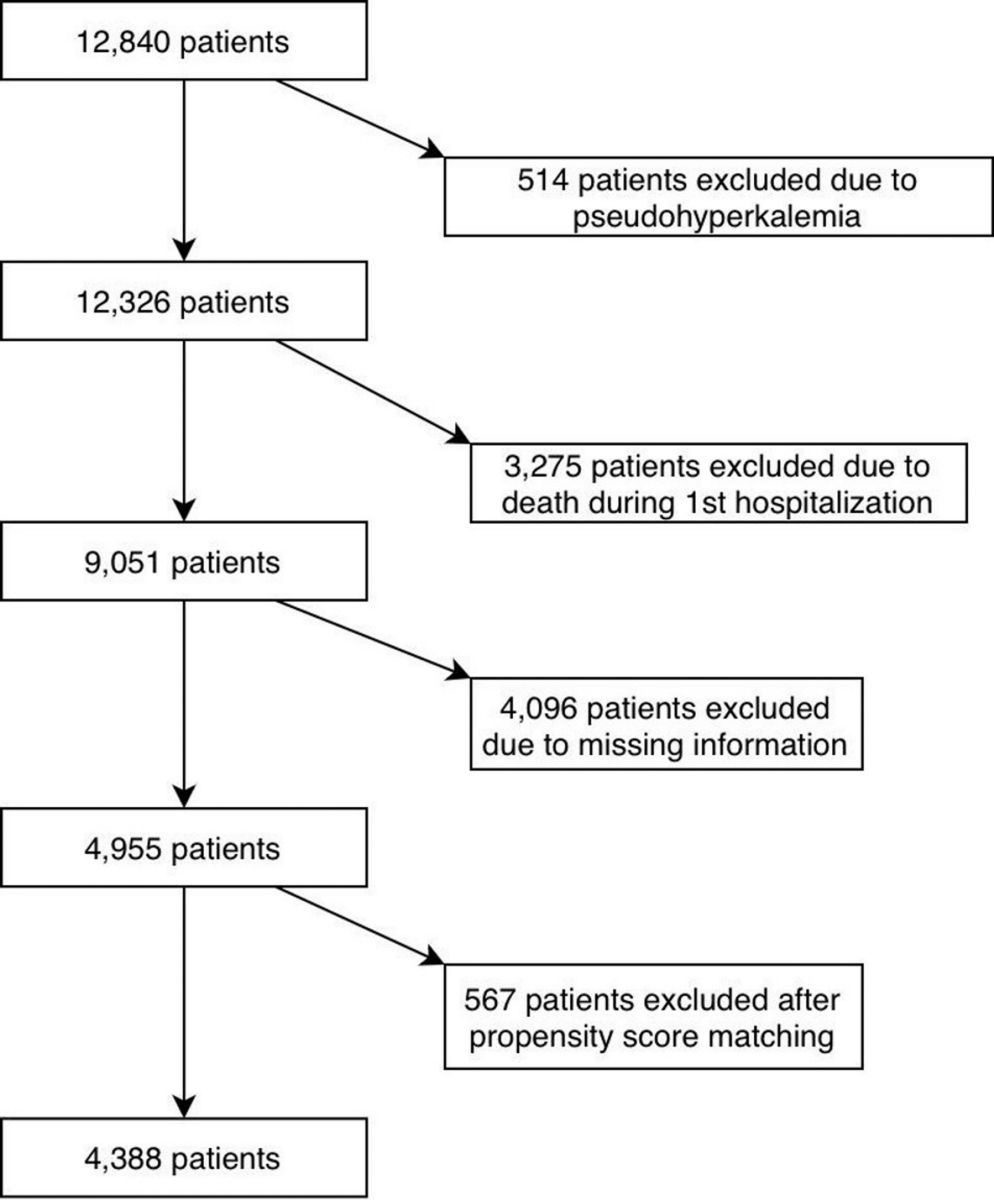
Flow chart of the patients included in this study

A total of 12,840 patients were admitted to Tel Aviv Medical Center for hyperkalemia while receiving chronic RASi therapy during the study period. After excluding 514 cases of pseudohyperkalemia, 12,326 patients with confirmed hyperkalemia were included in the final cohort (Figure 1). The cohort had a mean age of 75 ± 13 years, and 57% were male. Most admissions occurred in internal medicine departments (72.4%), followed by surgical departments (20%) and the intensive care unit (ICU, 7.2%). Among patients treated chronically with RASi, 58.6% were receiving ACEi, and 41.4% were prescribed ARBs.

The mean Charlson comorbidity index was 4 ± 3. Common comorbidities included DM (28.6%), CKD (21.7%), CHF (12.4%), and chronic obstructive pulmonary disease (COPD; 10.5%). The median serum potassium level at presentation was 5.7 mEq/L, and the median serum creatinine level was 1.6 mg/dL. During the first hospitalization, 26.6% of patients died, and 14.6% required acute dialysis. The median time to death was 48 days, and the median time to initiation of dialysis was 14 days. Patients who died during the index hospitalization represented a clinically complex and severely ill population, characterized by advanced age (mean 80 ± 11 years) and multiple comorbidities, including DM in 29.5%, CHF in 14.9%, CKD in 18.5%, and malignancy in 14.9% of patients. This mortality rate reflects the broad mix of cases over the 15-year study period, which included critically ill patients, advanced comorbidities, and acute presentations.

At subsequent hospitalizations, we examined the presence of RASi therapy in outpatient medication lists, as a secondary analysis, following discharge from their first admission. Medication data were available for 4,441 patients who survived their first hospitalization, were readmitted, and had documented outpatient medication lists. Among these patients, 31.8% (n = 1,411) continued RASi therapy. Continuation rates were higher among patients who were prescribed RASi at discharge compared with those who were not (58.6% (n = 827) vs. 41.1% (n = 584); odds ratio, 1.63; 95% CI, 1.44-1.85; P < .001).

A total of 9,051 patients survived the first hospitalization. Three of them had missing data, so the cohort included 9,048 patients (data are presented in Table 1). Among them, 4,096 were excluded due to incomplete hospitalization data, leaving 4,955 patients eligible for longitudinal analyses (Table 1). After PSM, 4,388 patients remained in the final analytic cohort (Figure 1).

**Table 1:** Characteristics and outcomes of patients prescribed RASi at discharge versus those not prescribed RASi in the entire cohort of survivors of the first hospitalization A total of 9048 patients were included in this analysis. Three patients were not included due to sealed medical file and no information. Values are presented as mean (standard deviation) for normally distributed continuous variables (using t-test statistics), median (interquartile range) for non-normally distributed continuous variables (using Mann-Whitney test), and number (percentage) for categorical variables (using Chi-square test). Corresponding p-values are derived from univariable analyses comparing patients prescribed versus those not prescribed renin–angiotensin system inhibitors (RASi) at discharge. Outcomes include all-cause mortality and initiation of chronic dialysis following discharge. Time-to-event outcomes are reported as days from the reference time point (using the log-rank test). RASi: Renin-angiotensin system inhibitors; MI: Myocardial infarction; CHF: Congestive heart failure; PVD: Peripheral vascular disease; CVA: Cerebrovascular accident; COPD: Chronic obstructive pulmonary disease; CTD: Connective tissue disease; PUD: Peptic ulcer disease; T2DM: Type 2 diabetes mellitus; CKD: Chronic kidney disease; IQR: Interquartile range.

	Before Propensity-Matched Analysis			
N = 9048	Prescribed RASi; N = 3458, N (%)	Not Prescribed RASi; N = 5590, N (%)	P-Value	Test Statistics
Gender (Male)	2020 (58.4)	3253 (58.2)	p = 0.8	Chi-square test
Age (years), mean ± SD	73.2 ± 13	73 ± 13.5	p = 0.5	t-test
Charlson score, median (IQR)	6 (5-8)	6 (5-8)	p < 0.001	Mann-Whitney (Wilcoxon rank-sum)
History of MI	271 (7.8)	260 (4.7)	p < 0.001	Chi-square test
CHF	509 (14.7)	532 (9.5)	p < 0.001	Chi-square test
PVD	259 (7.5)	333 (6)	p = 0.004	Chi-square test
CVA	359 (10.4)	530 (9.5)	p = 0.2	Chi-square test
Dementia	35 (1)	51 (0.9)	p = 0.6	Chi-square test
COPD	408 (11.8)	505 (9)	p < 0.001	Chi-square test
CTD	68 (2)	92 (1.6)	p = 0.3	Chi-square test
PUD	55 (1.6)	66 (1.2)	p = 0.1	Chi-square test
Liver disease	128 (3.7)	204 (3.6)	p = 0.6	Chi-square test
T2DM	1500 (43.4)	1854 (33.2)	p < 0.001	Chi-square test
Hemiplegia	6 (0.2)	20 (0.4)	p = 0.1	Chi-square test
CKD	845 (24.4)	1218 (21.8)	p = 0.004	Chi-square test
Potassium (mmol/L), median (IQR)	5.7 (5.6-6)	5.7 (5.6-6)	p < 0.001	Mann-Whitney (Wilcoxon rank-sum)
Creatinine (mg/dL), median (IQR)	1.6 (1.1-2.4)	1.5 (1.1-2.5)	p = 0.09	Mann-Whitney (Wilcoxon rank-sum)
Outcomes				
Death following discharge	565 (16.3)	1020 (18.2)	p < 0.001	Chi-square test
Time to death (days), median (IQR)	264 (76-432)	150 (51-362)	p < 0.001	Log-rank test
Chronic dialysis initiation	94 (2.7)	147 (2.6)	p = 0.3	Chi-square test
Time to dialysis initiation (days), median (IQR)	247 (1-1183)	31 (1-815)	p < 0.001	Log-rank test

The baseline characteristics and outcomes of patients prescribed RASi therapy at discharge versus those who were not prescribed RASi therapy post-hyperkalemia are presented in Table 2. Patients who were prescribed RASi therapy exhibited a higher prevalence of comorbidities, including CHF (17.1% vs. 12.5%, p < 0.001), COPD (12.9% vs. 10.8%, p = 0.022), type 2 diabetes mellitus (T2DM) (46.7% vs. 37.7%, p < 0.001), and a history of MI (8.7% vs. 5.5%, p < 0.001) (Table 2).

**Table 2:** Characteristics of patients prescribed versus those not prescribed RASi therapy at discharge: unmatched and propensity score-matched analyses Balance between groups was assessed using SMDs, with values < 0.1 considered indicative of negligible imbalance. CI: Confidence interval; RASi: Renin-angiotensin system inhibitors; MI: Myocardial infarction; CHF: Congestive heart failure; PVD: Peripheral vascular disease; CVA: Cerebrovascular accident; COPD: Chronic obstructive pulmonary disease; CTD: Connective tissue disease; PUD: Peptic ulcer disease; T2DM: Type 2 diabetes mellitus; CKD: Chronic kidney disease; SD: Standard deviation; IQR: Interquartile range; SMD: Standardized mean difference.

	Before Propensity Score-Matched Analysis			Following Propensity Score-Matched Analysis		
	Prescribed RASi; N = 2194, N (%)	Not Prescribed RASi; N = 2761, N (%)	SMD	Prescribed RASi; N = 2194, N (%)	Not Prescribed RASi; N = 2194, N (%)	SMD
Age, years, mean ± SD	73 ± 13	72 ± 14	0.065	73 (13)	73 (13)	0.014
Gender (Male)	1256 (57.2)	1704 (61.7)	0.091	1256 (57.2)	1246 (56.8)	0.009
Charlson score, mean (SD)	6.7 (2.1)	6.5 (2.3)	0.075	6.7 (2.1)	6.6 (2.2)	0.046
History of MI	191 (8.7)	153 (5.5)	0.123	191 (8.7)	148 (6.7)	0.073
CHF	375 (17.1)	344 (12.5)	0.131	375 (17.1)	321 (14.6)	0.067
PVD	187 (8.5)	202 (7.3)	0.045	187 (8.5)	157 (7.2)	0.051
CVA	254 (11.6)	309 (11.2)	0.012	254 (11.6)	240 (10.9)	0.020
Dementia	23 (1)	31 (1.1)	0.007	23 (1)	26 (1.2)	0.013
COPD	284 (12.9)	298 (10.8)	0.067	284 (12.9)	247 (11.3)	0.052
CTD	47 (2.1)	52 (1.9)	0.018	47 (2.1)	43 (2)	0.013
PUD	43 (2)	33 (1.2)	0.061	43 (2)	27 (1.2)	0.058
Liver disease	98 (4.5)	126 (4.6)	0.005	98 (4.5)	92 (4.2)	0.013
T2DM	1024 (46.7)	1042 (37.7)	0.182	1024 (46.7)	949 (43.3)	0.069
Hemiplegia	6 (0.3)	9 (0.3)	0.010	6 (0.3)	5 (0.2)	0.009
CKD	633 (28.9)	852 (30.9)	0.044	633 (28.9)	625 (28.5)	0.008
Potassium (mmol/L), median (IQR)	5.75 (5.6-6.0)	5.73 (5.6-6.0)	0.119	5.75 (5.6-6.0)	5.75 (5.6-6.0)	0.072
Severe hyperkalemia, potassium > 6.5 mmol/L	182 (8.3)	169 (6.1)	0.084	182 (8.3)	150 (6.8)	0.055
Creatinine (mg/dL), median (IQR)	1.74 (1.23-2.85)	1.81 (1.2-3.67)	0.137	1.74 (1.23-2.85)	1.72 (1.16-2.97)	0.005

Following PSM, prescription of RASi at discharge was associated with a significantly lower risk of mortality (HR, 0.62; 95% CI, 0.55-0.71; p < 0.001). Patients in the RASi group also had a higher risk of chronic dialysis initiation (HR, 1.57; 95% CI, 1.23-1.99; p = 0.002) (Table 3). Median time-to-event analyses, presented for descriptive purposes only, showed longer time to death among patients prescribed RASi (216 (84-433) vs. 154 (55-360) days) and a shorter time to dialysis initiation (141 (36-348) vs. 167 (50-365) days).

**Table 3:** Association between RASi prescription at discharge and clinical outcomes in the propensity score-matched cohort Hazard ratios (HRs) and 95% confidence intervals (CIs) were estimated using Cox proportional hazards models in the propensity score-matched cohort. All models, including chronic dialysis initiation, were adjusted for age and gender. Time-to-event outcomes were analyzed from the date of first hospital discharge to the occurrence of the event or censoring. RASi: Renin-angiotensin system inhibitors.

	Prescribed RASi; N = 2194, N (%)	Not Prescribed RASi; N = 2194, N (%)	Hazard Ratio (Confidence Interval)	P-Value
Death following discharge	401 (18.3)	642 (29.3)	0.62 (0.55-0.71)	<0.001
Chronic dialysis	167 (8.2)	111 (5.7)	1.57 (1.23-1.99)	0.002

**Figure 2:**
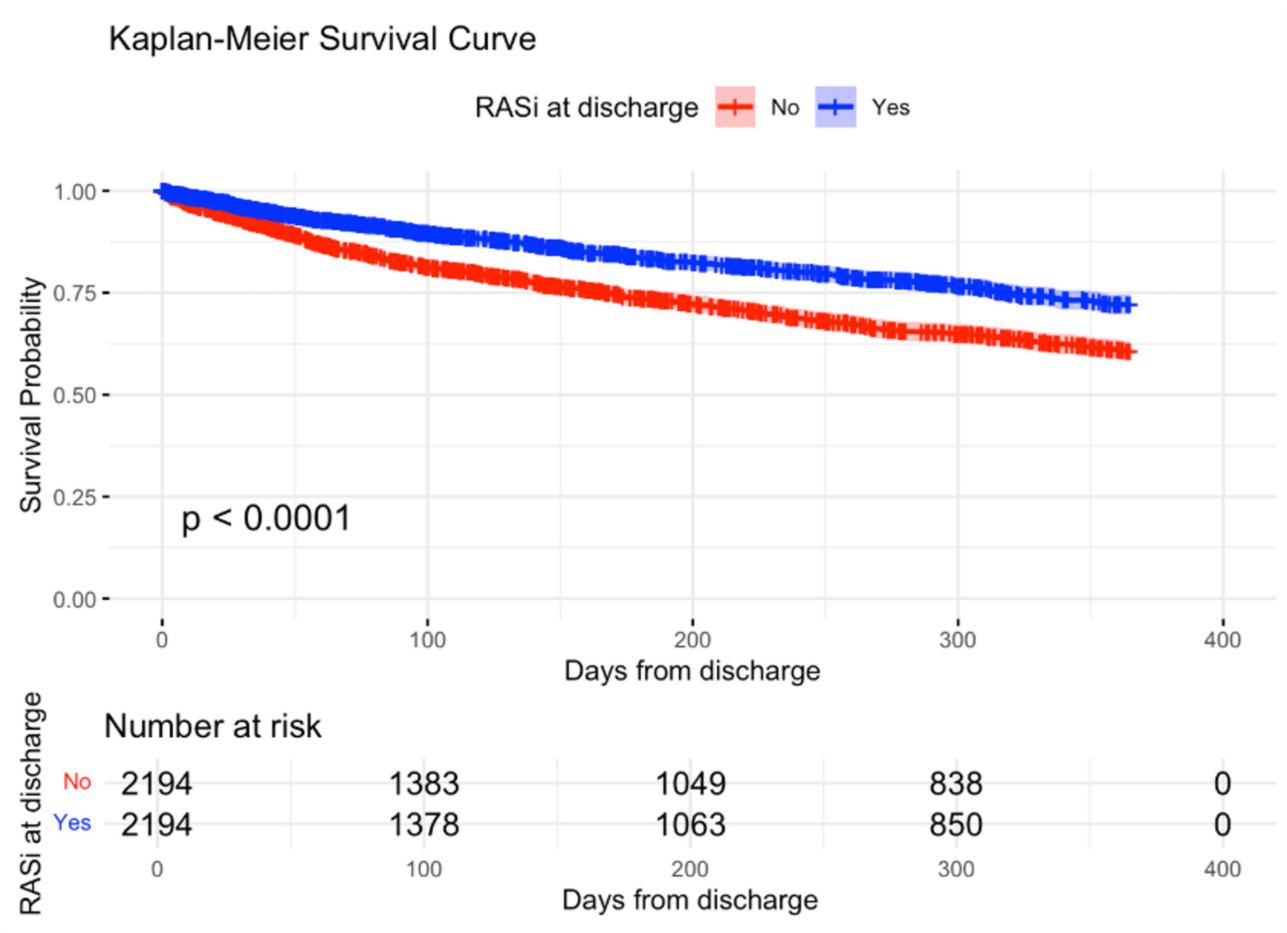
Kaplan-Meier survival curves for the propensity score-matched cohort for one year Time-to-event was calculated from hospital discharge to death. Patients prescribed RASi at discharge demonstrated improved survival compared with those not prescribed RASi. Survival distributions were compared using the log-rank test (p < 0.001). RASi: Renin-angiotensin system inhibitors.

Log-rank analysis revealed a statistically significant difference in survival in the first year after hospitalization between the two groups (χ² = 56.7, df = 1, p < 0.001). Patients in the RASi-prescribed group demonstrated better survival outcomes compared with those in the non-prescribed group (Figure 2).

In the propensity score-matched cohort, a Fine-Gray competing-risk regression analysis treating death before dialysis initiation as a competing event demonstrated that treatment remained independently associated with dialysis initiation (sHR, 1.10; 95% CI, 1.04-1.16; p = 0.0016).

**Figure 3:**
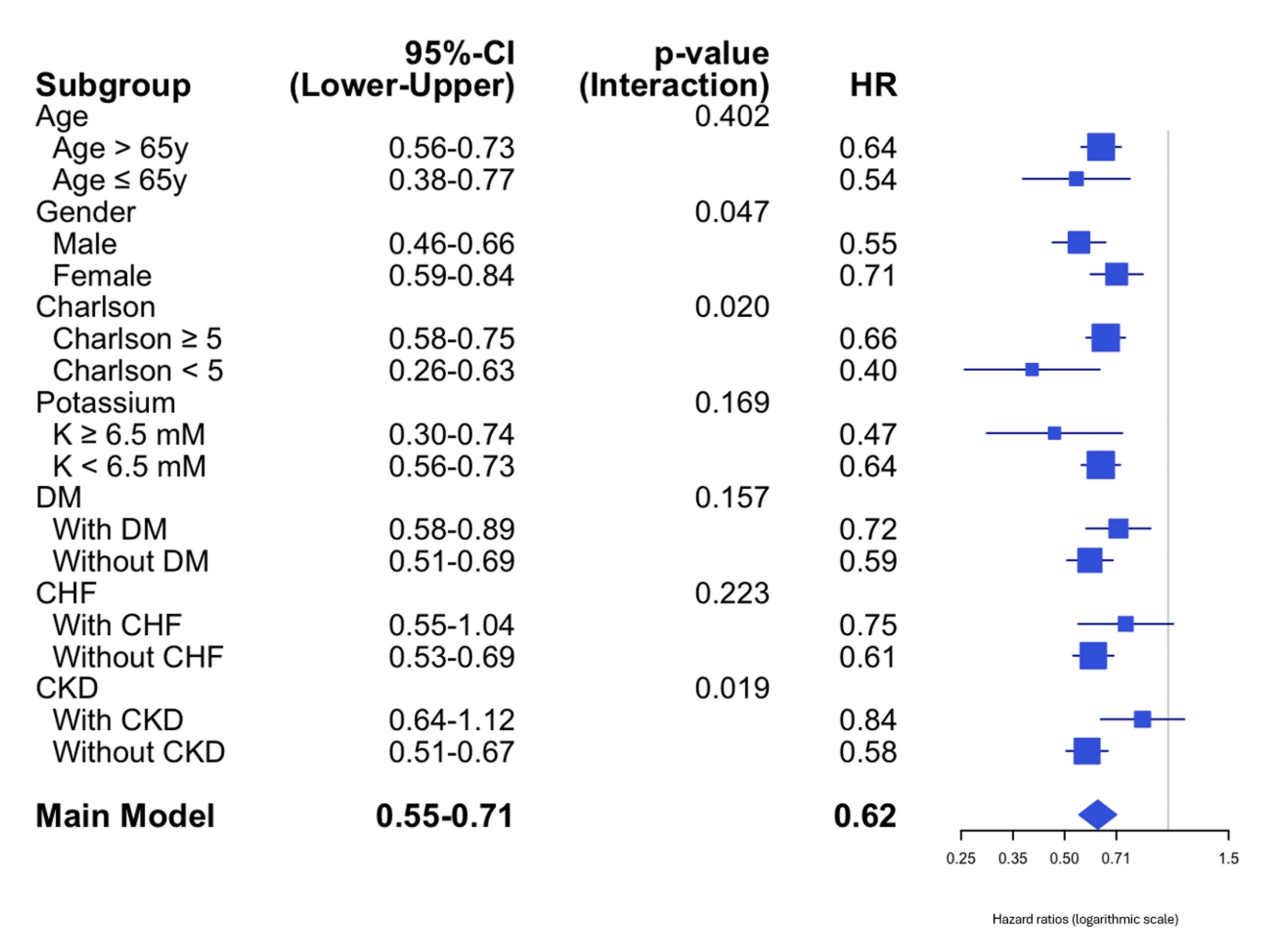
Subgroup analyses of the association between RASi prescription at discharge and one-year mortality in the propensity score-matched cohort Forest plot showing hazard ratios (HRs) and 95% confidence intervals (CIs) for the association between RASi prescription and mortality across predefined subgroups. HRs < 1 indicate a lower risk of death associated with RASi prescription. Interaction p-values were derived from Cox proportional hazards models including interaction terms between treatment group and subgroup variables. The x-axis represents HRs on a logarithmic scale. RASi: Renin-angiotensin system inhibitors; CKD: Chronic kidney disease; CHF: Congestive heart failure; DM: Diabetes mellitus; K⁺: Potassium.

Next, we performed a subgroup analysis for RASi therapy prescription versus discontinuation (Figure 3). Subgroup analyses largely confirmed the consistency of this association across most clinical subgroups, including patients with DM, younger and older adults, both genders, and all levels of hyperkalemia, including K > 6.5 mEq/L. We observed significant interactions for gender, comorbidity burden, and CKD status, indicating heterogeneity in the magnitude of the association between RASi prescription at discharge and mortality across these strata. No significant interactions were observed for age, potassium level, diabetes, or heart failure (Figure 3). While some subgroups (e.g., patients with CKD or CHF) showed wider CIs crossing 1.0, reflecting reduced statistical power, no subgroup suggested harm from prescribing the therapy.

## Discussion

In this study, we evaluated the association of morbidity and mortality with RASi prescription at discharge following hospitalization with a hyperkalemia event. Mortality during the index hospitalization was substantial, with approximately 26% of patients dying during hospitalization, reflecting the severe clinical condition and high comorbidity burden of this patient population. We further assessed survival and the initiation of dialysis, dividing the patients into two groups: those who were prescribed RASi therapy after their first admission and those who were not. Notably, patients who were prescribed RASi therapy had a higher burden of comorbidities, as reflected by significantly higher Charlson scores. Following PSM, mortality was significantly lower in the RASi prescription cohort. In the multivariable Cox proportional hazards models, prescription of RASi at discharge was associated with lower mortality (HR, 0.62; 95% CI, 0.55-0.71; P < .001). In contrast, the risk of initiating chronic dialysis was higher among patients prescribed RASi (HR, 1.57; 95% CI, 1.23-1.99; P = .002). These findings highlight the potential clinical implications of RASi prescription decisions at discharge, even in the presence of RASi-associated hyperkalemia, and documentation of these medications in discharge prescriptions.

The importance of prescribing the correct medication at discharge from hospitalization has already been tested in previous trials. In a study, HOPE (Heart Outcomes Prevention Evaluation) Study Investigators examined the relationship between hospital readmissions, mortality, and potentially inappropriate prescribing of medications and found that errors in medication prescription for older adults discharged from the hospital are significantly associated with repeated hospital admissions and mortality [15]. In the current study, prescribing of RASi therapy at discharge was associated with a significant delay in post-discharge mortality. These results are consistent with findings from studies such as the SOLVD trial and HOPE study, which demonstrated the benefits of RASi for survival in patients with heart failure and diabetes, respectively [15,16]. Similar results were presented in a study by Beusekamp et al., which demonstrated that compared with constant doses, down-titration or discontinuation of ACEi and ARBs was associated with a higher 180-day mortality [17]. Decreased mortality with regard to RASi therapy in CKD patients was also demonstrated in our study. These findings align with prior research by Qiao et al., which established that the absence of ACEi or ARB therapy is associated with an increased risk of mortality in CKD patients [12].

Patients who were prescribed RASi therapy had a significantly higher risk of progressing to chronic dialysis. However, this finding should be interpreted with caution. RASi treatment is well established for its reno-protective and cardioprotective benefits, particularly in patients with diabetic nephropathy or heart failure. The observed association between RASi prescription and increased dialysis risk may therefore reflect survivor bias or confounding by indication. Patients who remain on RASi therapy often live longer and therefore remain at risk for kidney disease progression for a longer time, ultimately increasing the likelihood of eventually requiring ​​​​​dialysis rather than causing kidney deterioration directly [15,16]. This phenomenon is supported by the HOPE study, which demonstrated that RASi therapy, specifically ramipril, was associated with lower mortality and cardiovascular events in patients with diabetes, thereby prolonging life expectancy. As patients live longer, the natural progression of CKD has more time to run its course, increasing the likelihood of eventually reaching dialysis [15]. Nevertheless, the association between discharge RASi prescription and chronic dialysis initiation remained significant after adjustment for death as a competing risk using a Fine-Gray regression model, supporting the robustness of the observed association. Another possible explanation for this observation is that hyperkalemia itself has been associated with an increased risk of kidney replacement therapy. Therefore, the higher incidence of chronic dialysis observed among patients discharged on RASi therapy may reflect both the underlying severity of renal disease and longer survival, rather than a direct adverse effect of RASi therapy [18-20].

The significant association between CHF and prescribed RASi therapy reflects the critical role of RASi in the management of heart failure. RASi therapy is associated with improved survival, fewer hospitalizations, and attenuation of cardiac remodeling [16]. Our findings align with those of Rosano et al., who reported widespread RASi use in heart failure patients (92.2% for ACEi) but noted that less than one-third were prescribed guideline-recommended target doses. While hyperkalemia was cited as a reason for non-use or dose reductions in 8.5% of cases for ACEi/ARBs, our findings showed that clinicians often prescribed RASi therapy even in the presence of severe hyperkalemia [9]. This discrepancy may reflect increased reliance on proactive hyperkalemia management strategies, such as potassium binders and dietary interventions, to maintain therapy while mitigating risks [21]. Both findings emphasize the need for improved strategies to optimize RASi dosing, highlighting the challenge of balancing reno-protective and cardioprotective benefits against the risks of adverse effects in patients with CHF​​​​​​.

Patients with CKD are at higher risk of hyperkalemia, which might prevent continuation of RASi therapy [9], as demonstrated by Rosano et al. Among patients with CKD, RASi were prescribed at the target guideline-recommended dose in only 19% to 26% of patients, prescribed at submaximal doses in 58% to 65% of patients, and discontinued during follow-up in 14% to 16% of patients. This highlights the challenge of balancing RASi’s reno-protective benefits against risks like hyperkalemia, emphasizing the need for proactive management strategies to optimize therapy in patients with CKD​​​​​​ [9].

In recent years, new oral potassium binders have been approved for the treatment of hyperkalemia secondary to chronic therapy with RASi, thereby allowing continuation of these medications [22]. The use of patiromer (Veltassa) in clinical trials of up to one year has shown clinically meaningful potassium reductions, sustained normokalemia, high tolerability, and a lack of major serious adverse events [23]. Sodium zirconium cyclosilicate (Lokelma) is a newer drug approved for the treatment of hyperkalemia, which can also be used in acute hyperkalemia [24]. These new treatment options, along with a low potassium diet, thiazide diuretics, and the newer sodium-glucose cotransporter 2 (SGLT2) inhibitors, provide options for treatment of life-threatening hyperkalemia due to RASi therapy and may help prevent discontinuation of these medications [25]. Current guidelines and expert recommendations emphasize that, when RASi therapy remains clinically indicated, hyperkalemia should be actively managed rather than prompting discontinuation of RASi therapy. This may include close monitoring of serum potassium and renal function during hospitalization and after discharge, reassessment of contributing medications and dietary factors, and consideration of newer potassium binders such as patiromer or sodium zirconium cyclosilicate to facilitate ongoing guideline-directed therapy [3,26,27]. Hospitalization may provide a unique opportunity for continuation or reinitiation of RASi therapy, as serum potassium levels and kidney function can be closely monitored in a controlled setting. This allows timely adjustment of treatment and management of recurrent hyperkalemia, potentially facilitating safe discharge on guideline-directed RASi therapy [25-27].

Despite the strengths of our study, several limitations should be acknowledged. As a retrospective cohort study, the analysis relied on pre-existing medical records and databases, limiting control over potential confounding variables due to incomplete or inconsistent data capture. To mitigate this limitation, several strategies were implemented to enhance validity, including Cox regression analyses to adjust for known confounders and PSM to reduce baseline differences between groups. This study lacked direct information regarding patient adherence or post-discharge management and, therefore, classified exposure according to discharge prescription records, which reflect physician treatment intent rather than confirmed long-term medication adherence. Because the study aimed to evaluate the impact of hospitalization with hyperkalemia on RASi management decisions, exposure was defined at hospital discharge. Nevertheless, immortal time bias cannot be completely excluded in this observational design. In addition, subsequent outpatient RASi use, as determined by treating physicians following discharge, was not captured. Accordingly, the findings should be interpreted as reflecting in-hospital discontinuation or non-reinitiation of RASi therapy. Given the observational nature of the study, the findings should be interpreted as associations rather than causal effects. Information bias arising from inaccuracies or variations in data recording was minimized by validation of a representative sample of 900 cases against the Chameleon electronic medical record system.

An additional limitation is that patients with incomplete hospitalization data were excluded from the analysis. Although these exclusions were necessary to ensure reliable propensity score estimation and outcome assessment, they may have introduced selection bias and may limit the generalizability of the findings. Important clinical variables such as race, socioeconomic status, proteinuria, recurrent hyperkalemia episodes, outpatient potassium monitoring, accurate medication dosing, severity of acute illness, and cause of death data were unavailable for a substantial proportion of patients and, therefore, could not be reliably incorporated into the analysis. Blood pressure measurements were also not included because patients had multiple recordings during hospitalization with substantial intra-patient variability, even during short hospital stays, limiting the ability to derive standardized and clinically meaningful variables for adjustment.

Although the estimated glomerular filtration rate (eGFR) is an important marker of kidney function, many patients experienced acute kidney injury during hospitalization, limiting the interpretability and reliability of eGFR calculations in this setting. Therefore, serum creatinine levels were used instead in the matching model. In addition, information regarding race and ethnicity was not consistently available in the medical records and, therefore, could not be incorporated into the analyses. Residual confounding may persist despite PSM and multivariable adjustment, as patients discharged on RASi therapy may have differed in overall clinical status or physician-assessed prognosis in ways not fully captured by the available variables. Finally, death may have acted as a competing risk for chronic dialysis initiation, potentially influencing the observed association between RASi prescription and progression to chronic dialysis. Similarly, patients who survived longer may have had a greater opportunity to progress to chronic dialysis, and therefore dialysis-related findings should be interpreted cautiously. Subgroup analyses were exploratory in nature and were not adjusted for multiple testing; accordingly, these findings should also be interpreted cautiously. Finally, this was a single-center study conducted within the Israeli healthcare system; therefore, generalizability to other healthcare settings and patient populations may be limited.

## Conclusions

In conclusion, RASi prescription at discharge was associated with improved survival but increased dialysis initiation, highlighting the need for individualized post-hyperkalemia management.
